# Aberrant methylation in neurofunctional gene serves as a hallmark of tumorigenesis and progression in colorectal cancer

**DOI:** 10.1186/s12885-023-10765-x

**Published:** 2023-04-06

**Authors:** Xuan Li, Du Cai, Yaoyi Huang, Yumo Xie, Dingcheng Shen, Ze Yuan, Xiaoxia Liu, Meijin Huang, Yanxin Luo, Huichuan Yu, Xiaolin Wang

**Affiliations:** 1 Department of Colorectal Surgery, The Sixth Affiliated Hospital, Sun Yat-Sen University, Guangzhou, Guangdong China; 2 Guangdong Institute of Gastroenterology, Guangzhou, Guangdong China; 3 Department of General Surgery, The Sixth Affiliated Hospital, Sun Yat-Sen University, Guangzhou, Guangdong China; 4 Guangdong Provincial Key Laboratory of Colorectal and Pelvic Floor Diseases, The Sixth Affiliated Hospital, Sun Yat-Sen University, Guangzhou, Guangdong China

**Keywords:** Colorectal cancer, DNA methylation, Prognosis, Biomarker, *RIMS2*, *KRAS*

## Abstract

**Background:**

DNA methylation is one of the most promising biomarkers in predicting the prognosis of colorectal cancer (CRC). We aimed to develop a DNA methylation biomarker that could evaluate the prognosis of CRC.

**Methods:**

A promising DNA methylation biomarker was developed by hypermethylated genes in cancer tissue that were identified from Illumina EPIC methylation arrays. A cohort comprising 30 pairs of snap-frozen tumor tissue and adjacent normal tissue was used for correlation analysis between the methylation and expression status of the marker. The other cohort comprising 254 formalin-fixed paraffin-embedded (FFPE) tumor tissue from 254 CRC patients was used for prognosis analysis.

**Results:**

Regulating synaptic membrane exocytosis 2 (*RIMS2*) was hypermethylated and lowly expressed in CRC comparing to adjacent normal tissue. Hypermethylation of *RIMS2* in CRC was correlated with less frequent *KRAS* mutant and high differentiation. *RIMS2* promoter methylation showed independent predictive value for survival outcome (*P* = 0.015, HR 1.992, 95% CI [(1.140–3.48)]), and a combination of *RIMS2* methylation with *KRAS* status could predict prognosis better.

**Conclusions:**

*RIMS2* is frequently hypermethylated in CRC, which can silence the expression of *RIMS2*. *RIMS2* methylation is a novel biomarker for predicting the prognosis of CRC.

## Background

Colorectal cancer (CRC) is one of the most prevalent malignant tumors around the world, accounting for nearly 10.2% of new cancer cases and 9.2% of total cancer deaths globally [1, 2]. With increasing use of prognostic biomarkers, molecularly stratified therapy for CRC has been gradually improved [3, 4]. However, the identification of stable and easily detectable biomarkers remains a challenge.

Among these biomarkers, epigenetic alterations, especially DNA methylation, is one of the most promising candidates in clinical practice [5–7]. DNA methylation is a covalent modification, which always occurs on cytosine nucleotides and exclusive in the context of CpG site [8]. Aberrant methylation of CpG islands in gene promoter is associated with silencing of tumor suppressor gene, which facilitates tumorigenesis and development [9]. As a biomarker, DNA methylation has several advantages to qualify them for broad use, like high stability and repeatability [10]. Previous studies have excavated some DNA methylation biomarkers for prognosis of CRC, such as CIMP, MGMT, and SEPT9. However, reliable DNA methylation biomarkers for clinical practice of CRC are still limited [11].

To identify new DNA methylation biomarkers, we have conducted a genome-wide screen for hypermethylated genes in cancer tissue by using Infinium MethylationEPIC (EPIC) BeadChip. Among all, regulating synaptic membrane exocytosis 2 (*RIMS2*) is one of the top candidate genes. *RIMS2*, also known as *RIM2*, codes for a presynaptic active zone protein with multidomain, including Rim2α, Rim2β, and Rim2γ [12]. Rim2α, the full-length form of Rim2, is composed of an N-terminal Zn^2+^ -finger domain, a central PDZ and C2A domains, and a C-terminal C2B domain [13]. Rim2α participates in Ca^2+^ -dependent neurotransmitter release from synaptic vesicles by interacting with Rab3 [14, 15]. Rim2α also interacts with Munc13-1 [16], Rab8 [17], cAMP-GEFII [18], and ELKS [19]. Rim2α is expressed mainly in endocrine and neuroendocrine cells such as pancreatic β cells, pituitary, and adrenal gland [13]. Several studies have suggested that abnormal expression of *RIMS2* may involve in the development of cancer [20, 21]. However, there is no relative study between *RIMS2* and CRC.

In this study, we determined the methylation and expression status of *RIMS2* in CRC and investigated the prognostic utility of *RIMS2* promoter methylation in CRC.

## Methods

### Study cohort and data collection﻿

Two cohorts were used in this study. Cohort 1 comprises 30 pairs of snap-frozen tumor tissue and adjacent normal tissue from 30 CRC patients, which was used for correlation analysis between the methylation and expression status of *RIMS2* in CRC tissue. Cohort 2 comprises 254 formalin-fixed paraffin-embedded (FFPE) tumor tissue from 254 CRC patients who underwent radical surgery between 2009 and 2012 at the Sixth affiliated hospital of Sun Yat-Sen University [22, 23]. Cohort 2 was used for prognosis analysis. Only pathologically confirmed CRC cases were eligible. The following clinical materials were obtained from the Electronic Medical Record System: demographic characteristics (gender, age), tumor location, TNM cancer stage, MSI status, *KRAS* mutation, differentiation degree, preoperative serum CEA level, lymphovascular invasion, and perineural invasion. Polymerase chain reaction (PCR) based methods have been used to assess MSI status. The study was approved by the Medical Ethics Committee at the Sixth Affiliated Hospital of Sun Yat-sen University. Follow-up data were applied from a CRC database of the Sixth Affiliated Hospital of Sun Yat-sen University. The follow-up was censored in September 2018 with a median follow-up of 85.1 months.

### Cell culture and 5-aza-2’-deoxycytidine treatment

DLD1 and HCT116 CRCcolorectal cancer cell line purchased from American Type Culture Collection (ATCC) were cultured at proper medium at 37℃ with 5% CO_2_ following the manufacturer’s protocol. A demethylation drug 5-aza-2’-deoxycytidine (5-Aza, sigma, A3656) was used to treat the above CRC cell lines as previous study described [24].

### DNA extraction and quantitative methylation-specific PCR

Genomic DNA from snap-frozen cancer tissue and CRC FFPE was extracted by the QIAamp DNA Mini Kit (Qiagen, 51,306), and then sodium bisulfite conversion was completed by using the EZ DNA Methylation Kit (Zymo Research, D5002). *RIMS2* promoter methylation was measured using quantitative methylation-specific PCR (qMSP) as the previous study described [25, 26]. CpGenome Universal Methylated DNA Kit (Millipore, S7821) was used as a fully methylated positive control (M). CpGenome Universal Unmethylated DNA Set (Millipore, S7822) was used as the negative unmethylated control (U). Aluc4 was used as an internal reference. The specific sequence of primer and probe of *RIMS2* and Aluc4 used in this study were provided in Table 1. The methylation degree of the target locus was calculated by percentage methylated reference (PMR), which was calculated by dividing the *RIMS2*/Aluc4 ratio of a sample by the *RIMS2*/Aluc4 ratio of fully methylated control and multiplied by 100% [27].

**Table 1:** Primer and probe used for qMSP and RT-qPCR

*RIMS2* qMSP primer and probe	
Forward	GGGTTGAGGAGTGCGTTTCG
Backward	CGTCCCGACCTCCAATTCC
FAM probe	6FAM-AGGGTTGTGAAGTGAGCGG-MGB-NFQ
Aluc4 qMSP primer and probe	
Forward	GGTTAGGTATAGTGGTTTATATTTGTAATTTTAGTA
Backward	ATTAACTAAACTAATCTTAAACTCCTAACCTCA
FAM probe	6FAM-CCTACCTTAACCTCCC-MGB-NFQ
*RIMS2* qPCR primer	
Forward	GGTTCGGCTCCACCAAACAT
Backward	TTTCCTCTCCTCCTCCGTGA
*GAPDH* qPCR primer	
Forward	AGAAGGCTGGGGCTCATTTG
Backward	AGGGGCCATCCACAGTCTTC

qMSP quantitative methylation-specific PCR, RT-qPCR reverse transcription quantitative PCR, *RIMS2* regulating synaptic membrane exocytosis 2, *GAPDH* glyceraldehyde-3-phosphate dehydrogenase

### RNA extraction and quantitative real-time PCR

Total RNA extraction was done by using the UNlQ-10 Column Trizol Total RNA Isolation Kit (Sangon Biotech, B511321). Reverse transcription was conducted by the QuantiTect Reverse Transcription Kit (Qiagen,205,311). Reverse transcription‑quantitative PCR (RT-qPCR) was carried out with a QuantStudio 7 flex Real-Time PCR system and FastStart Essential DNA Green Master (Roche, 06,402,712,001) following the manufacturer’s protocol. Glyceraldehyde-3-phosphate dehydrogenase (*GAPDH*) was used as an internal control. Primers’ sequence of *RIMS2* and *GAPDH* used for RT-qPCR were listed in Table1. The relative mRNA expression level of *RIMS2* was calculated by the 2(^−△△CT^) method [28].

### Statistical analysis

Comparison of the *RIMS2* methylation and mRNA expression between cancer and adjacent normal tissue was done by paired Wilcoxon signed-rank test. The relationship between the DNA methylation and mRNA expression of *RIMS2* in cancer tissue was tested by Spearman’s Rank Correlation analysis. The optimal cutoff point for *RIMS2* methylation in prognosis analysis was determined by maximally selected rank statistic [29]. Based on this cutoff, patients in Cohort 2 were divided into methylation-low and methylation-high groups. For the comparison of baseline characteristics, Wilcox rank-sum test was applied for continuous variables, and the chi-square test was applied for discrete variables. The primary outcomes were disease-free survival (DFS). DFS was the length of time from radical resection to recurrence. Cox regression analysis and Kaplan–Meier survival curves with log-rank tests were used to compare DFS. The variables considered clinically relevant or reached the significance level of *P* < 0.1 in univariate Cox regression were entered into the multivariate Cox regression analysis. The comparison between predictive models was assessed using likelihood ratio (LR) and Akaike information criterion (AIC) in competing models. In general, the model with a lower AIC and a higher LR were considered a better option. The predictive nomogram was developed based on the best model. Calibration curves were plotted to explore the predictive accuracy of the nomogram. A receiver operating characteristic (ROC) curve was plotted to assess the discriminative ability of the nomogram. Discriminative ability was quantified with the area under the ROC curve (AUC). Two-tailed tests with *P* < 0.05 were considered statistically significant. All statistical analyses were conducted using SPSS (version 26.0) or R (version 3.6.0).

## Results

### *RIMS2* expression is prevalently silenced by promoter methylation in CRC

**Fig. 1:**
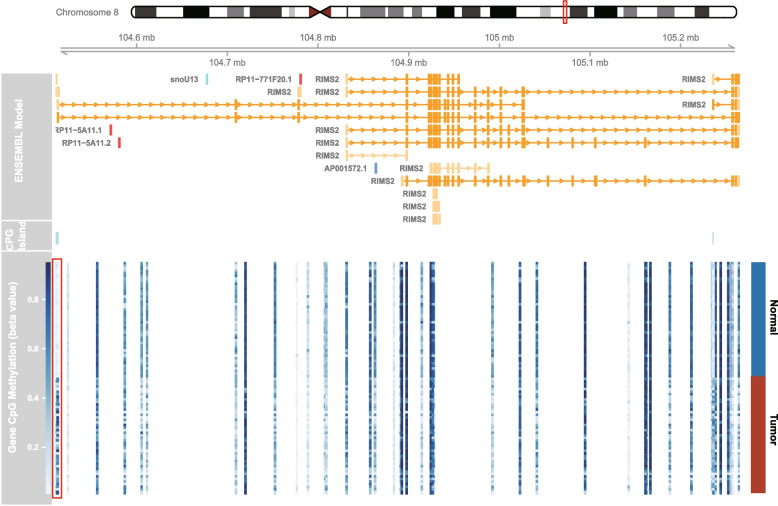
Schematic diagram of the differential methylation region in 48 pairs of cancer and normal tissue. The methylation values of each EPIC probe in CRC and normal tissue was shown as a heatmap. The differential methylation region with the most significant difference was marked by a red box and used for following qMSP, which was located at CpG island of *RIMS2*. *RIMS2*: regulating synaptic membrane exocytosis 2; qMSP: quantitative methylation-specific PCR

**Fig. 2:**
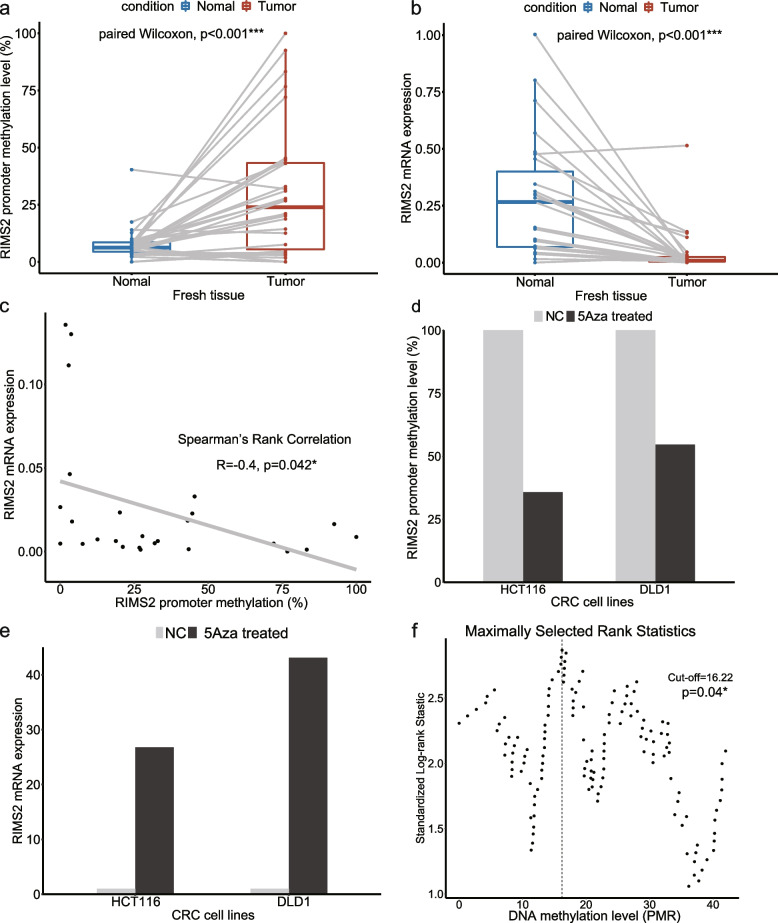
*RIMS2* expression is silenced by promoter hypermethylation in CRC. **a.** *RIMS2* promoter methylation level in paired fresh CRC and adjacent normal tissue. Y-axis showed the percentage methylated reference (PMR), which was used to calculate the methylation level. **b.** *RIMS2* mRNA expression level in paired fresh CRC and adjacent normal tissue. **c.** Correlation between *RIMS2* promoter methylation and mRNA expression in CRC tissue. **d.** *RIMS2* promoter methylation level in HCT116 and DLD1 CRC cell line before and after 5-Aza treatment. **e.** *RIMS2* mRNA expression level in HCT116 and DLD1 CRC cell line before and after 5Aza treatment. *P < 0.05; **f.** Maximally selected rank statistic was used to select the optimal cutoff point for *RIMS2* promoter methylation

By analyzing the data from Infinium MethylationEPIC (EPIC) BeadChip, *RIMS2* promoter CpG island was found to be significantly hypermethylated in CRC comparing to normal tissue (Fig. 1). The methylation level of *RIMS2* promoter was validated by qMSP in 30 cases of snap-frozen CRC and matched adjacent normal tissue. The methylation level of *RIMS2* was significantly higher in CRC tissue (Fig. 2a). *RIMS2* expression was proved to be suppressed in CRC tissue measured by quantitative real-time PCR (Fig. 2b). Spearman test and dot plot showed that *RIMS2* expression was negatively correlated with *RIMS2* promoter methylation (Fig. 2c). To further validate the regulation of *RIMS2* expression by *RIMS2* methylation, a demethylation reagent 5-Aza (5-aza-2’-deoxycytidine) was used to treat DLD1 and HCT116 cell lines. Restoration of *RIMS2* expression was found in DLD1 and HCT116 after 5-Aza treatment, with the decreasing of methylation level (Fig. 2d and e). These results suggested that *RIMS2* expression was silenced by promoter hypermethylation in CRC.

### Association of *RIMS2* methylation with clinicopathological, molecular and features in patients with CRC

254 formalin-fixed paraffin-embedded (FFPE) tumor tissue from CRC patients who underwent radical surgery were obtained for prognosis analysis. *RIMS2* methylation level in these patients’ samples was measured by qMSP as well. By using the maximally selected rank statistics in an R package called “Maxstat”, PMR of 16.22% was set as the optimal cutoff point for *RIMS2* methylation (Fig. 2f). Based on this cutoff, these 254 patients were split into methylation-low (*n* = 113) and methylation-high (*n* = 141) group. As shown in Table 2, *RIMS2* hypermethylation was correlated with *KRAS* mutation and differentiation. No significant association was found between *RIMS2* methylation and age, gender, tumor location, TNM stage, MSI, lymphovascular invasion, perineural invasion and preoperative serum CEA.

**Table 2:** Association of the *RIMS2* methylation with the baseline characteristics

Characteristics	Total	*RIMS2* promoter methylation		*P* value
		Low (*n* = 113)	High (*n* = 141)	
Age(median = 62 years)				0.832
< 62	115	52	63	
≥ 62	139	61	78	
Gender				0.389
Male	143	67	76	
Female	111	46	65	
Tumor location				0.664
Colon	131	60	71	
Rectum	123	53	70	
TNM stage				0.195
I-II	162	77	85	
III-IV	92	36	56	
MSI status				0.072
MSI	60	36	24	
MSS	145	67	78	
NA	49	10	39	
*KRAS* status				0.010*
Wild type	131	57	74	
Mutant	81	50	31	
NA	42	6	36	
Differentiation				0.035*
Poor	13	2	11	
Medium	132	56	76	
High	76	40	36	
NA	33	15	18	
Lymphovascular invasion				0.779
Negative	235	105	130	
Positive	17	7	10	
NA	2	1	1	
Perineural invasion				0.088
Negative	233	100	133	
Positive	19	12	7	
NA	2	1	1	
CEA				0.651
0-5 ng/ml	176	78	98	
> 5 ng/ml	63	30	33	
NA	15	5	10	

^*^P < 0.05; *RIMS2* regulating synaptic membrane exocytosis 2, *MSI* microsatellite instability, *MSS* microsatellite stability, *CEA* carcinoembryonic antigen, *NA* not available

### Prognostic value of *RIMS2* methylation in CRC

**Fig. 3:**
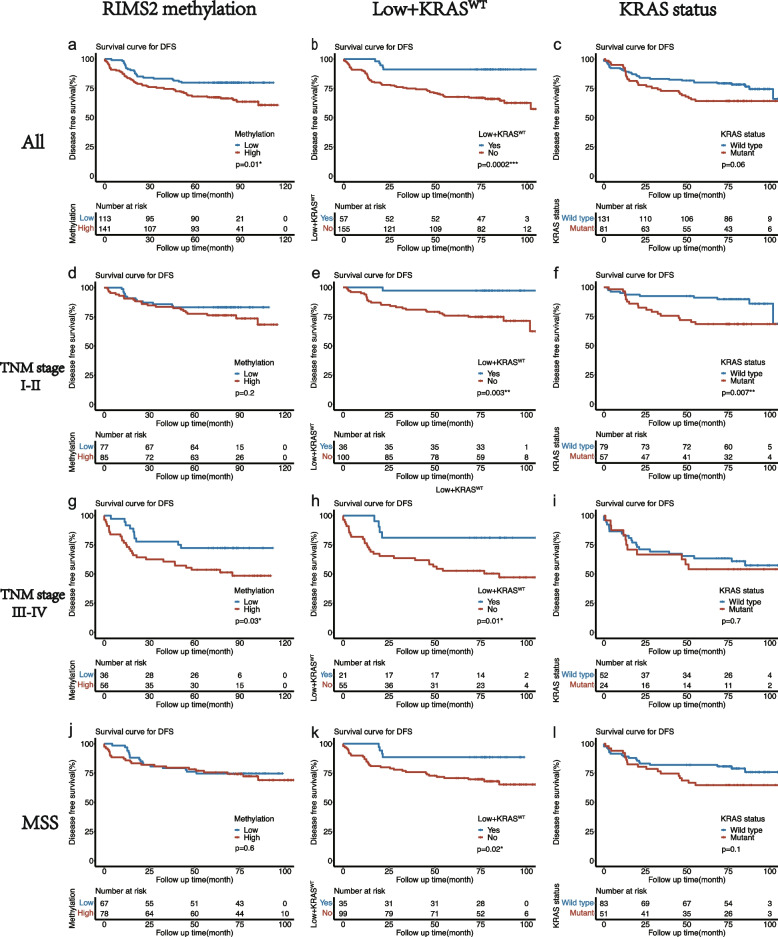
Kaplan–Meier curves of DFS in all patients and different subgroups. (**a** **d** **g** **j**). Kaplan-Meier survival curves of DFS of low *RIMS2* methylation group and high *RIMS2* methylation group in all patients, TNM stage I-II subgroup, TNM stage III-IV subgroup and MSS subgroup, respectively; (**b** **e** **h** **k**). Kaplan-Meier survival curves of DFS of Low + *KRAS*^WT^ group and no Low + *KRAS*^WT^ group in all patients, TNM stage I-II subgroup, TNM stage III-IV subgroup and MSS subgroup, respectively; (**c** **f** **i** **l**). Kaplan-Meier survival curves of DFS of *KRAS* mutant group and *KRAS* wild group in all patients, TNM stage I-II subgroup, TNM stage III-IV subgroup and MSS subgroup, respectively

Kaplan–Meier curve revealed a significantly poor DFS (*P* = 0.01) in the *RIMS2* hypermethylation group (Fig. 3a). Univariate analysis showed that older age, advanced TNM stage, lymphovascular invasion, preoperative higher CEA and *RIMS2* hypermethylation were associated with poor DFS (Table 3). To eliminate the influence of potential confounders, a multivariate Cox analysis was conducted. According to the multivariate analysis, older age, advanced TNM stage, *KRAS* mutation, lymphovascular invasion and *RIMS2* hypermethylation were associated with poor DFS (Table 3). Taken together, *RIMS2* methylation was independently associated with DFS (HR: 1.992 (1.140–3.479), P = 0.015) in CRC. Furthermore, we compared the prognostic value of *RIMS2* methylation in different subgroups. Kaplan–Meier curves showed that DFS was not significantly different in the TNM stage I-II subgroup (Fig. 3d) and MSS subgroup (Fig. 3j), only significantly different in the TNM stage III-IV subgroup (Fig. 3g).

**Table 3:** Cox proportional hazard analyses on DFS in patients with CRC

Variables	Univariate		Multivariate^a^		Multivariate^b^	
	HR(95%CI)	*P* value	HR(95%CI)	*P* value	HR(95%CI)	*P* value
Age		0.021*		0.014*		0.018*
< 62	1		1		1	
≥ 62	1.781(1.093–2.903)		2.102(1.165–3.790)		2.039(1.132–3.675)	
Gender		0.388				
Male	1					
Female	0.812(0.507–1.302)					
Tumor location		0.951				
Colon	1					
Rectum	1.015(0.641–1.606)					
TNM stage		0.001*		0.014*		0.016*
I-II	1		1		1	
III-IV	2.203(1.391–3.488)		1.616(1.103–2.360)		1.597(1.090–2.340)	
MSI status		0.675				
MSS	1					
MSI	1.127(0.645–1.970)					
*KRAS* mutation		0.065		0.004*		
Wild type	1		1			
Mutant	1.612(0.971–2.677)		2.273(1.306–3.950)			
Differentiation						
Poor	1					
Medium	0.632(0.249–1.604)	0.334				
High	0.518(0.193–1.388)	0.191				
Lymphovascular invasion		0.036*		0.001*		0.001*
Negative	1		1		1	
Positive	2.196(1.052–4.583)		3.711(1.67–8.245)		3.904(1.750–8.710)	
Perineural invasion		0.683				
Negative	1					
Positive	1.190(0.516–2.747)					
CEA		0.039*		0.252		0.274
0-5 ng/ml	1		1		1	
> 5 ng/ml	1.455(1.020–2.074)		1.281(0.838–1.950)		1.266(0.830–1.930)	
*RIMS2* methylation		0.011*		0.015*		
Low	1		1			
High	1.893(1.155–3.103)		1.992(1.140–3.479)			
Low + *KRAS*^WT^		0.001*				0.001*
Yes	1				1	
No	4.761(1.905–11.897)				4.795(1.893–12.145)	

^*^P < 0.05; *RIMS2* regulating synaptic membrane exocytosis 2, *MSI* microsatellite instability, *MSS* microsatellite stability, *CEA* carcinoembryonic antigen; Low + *KRAS*^WT^: patients with low *RIMS2* methylation and *KRAS* wild type; Multivariate^a^: including age, TNM stage, *KRAS* mutation, lymphovascular invasion, CEA and *RIMS2* methylation; Multivariate^b^: replacing *KRAS* status and *RIMS2* methylation with Low + *KRAS*^WT^, compared to Multivariate^a^

Next, we explored whether *RIMS2* methylation could further stratify *KRAS* wild and *KRAS* mutant patients in marker prediction assays. In survival analysis, patients were divided into different groups according to whether with low methylation and *KRAS* wild type at the same time (Patients with unknown *KRAS* status were excluded). As expected, patients with low methylation and *KRAS* wild type had a significantly better DFS not only in all patients (Fig. 3b) but also in the 3 subgroups (Fig. 3e, h, k). As for *KRAS* status, DFS was significantly different only in the TNM stage I-II subgroup (Fig. 3f), but not in all patients (Fig. 3c) and other subgroups (Fig. 3i, l). So Low + *KRAS*^WT^ (Patients with low *RIMS2* methylation and *KRAS* wild type) may be a better prognosis biomarker. Another multivariate COX analysis, including age, Low + *KRAS*^WT^, TNM stage, lymphovascular invasion and CEA, showed that Low + *KRAS*^WT^ was independently associated with DFS (Table 3).

### Comparison between different models

Model 3 had a lower AIC and a higher LR compared with model 1 (AIC: 764.44 s. 768.59; LR: 17.34 vs. 11.81, *P* = 0.013; Table 4). In the comparison between model 4 and 5, after *RIMS2* methylation was added, a lower AIC and a higher LR were observed (AIC: 530.90 vs. 526.91; LR: 24.61 vs. 30.60, *P* = 0.014; Table 4). These results showed that *RIMS2* methylation could increase the prognostic values of current prognostic panels. In the comparison between model 5 and model 6, after replacing *KRAS* status and *RIMS2* methylation with Low + *KRAS*^WT^, a lower AIC and a higher LR were observed (AIC: 526.91 vs. 520.83; LR: 30.60 vs. 34.68, *P* = 0.043; Table 4). The result showed that the Low + *KRAS*^WT^ could predict prognosis better, and model 6 was the best predictive model in the study.

**Table 4:** Comparison between different models

Models	N	AIC	LR	*P* value
Model 1	254	768.59	11.81	
Model 2	254	772.96	6.82	
Model 3	254	764.44	17.34	0.013^a^
Model 4	198	530.90	24.61	0.014^b^
Model 5	198	526.91	30.60	
Model 6	198	520.83	34.68	0.043^c^

*N* patient counts in each model, *AIC* Akaike information criterion value, *LR* likelihood ratio; Model 1 includes TNM stage; Model 2 includes *RIMS2* methylation

Model 1 includes TNM stage; Model 2 includes RIMS2 methylation; Model 3 includes TNM stage and *RIMS2* methylation; Model 4 includes age, TNM stage, *KRAS* mutation, lymphovascular invasion; Model 5 includes *RIMS2* methylation and all variables in Model 4; Model 6 replaces *KRAS* status and *RIMS2* methylation with Low + *KRAS*^WT^, compared to Model5; ^a^*P* values for the LR test in model 1 compared with model 3; ^b^*P* values for the LR test in model 4 compared with model 5; ^c^*P* values for the LR test in model 5 compared with model 6

### A nomogram for predicting DFS in CRC patients

**Fig. 4:**
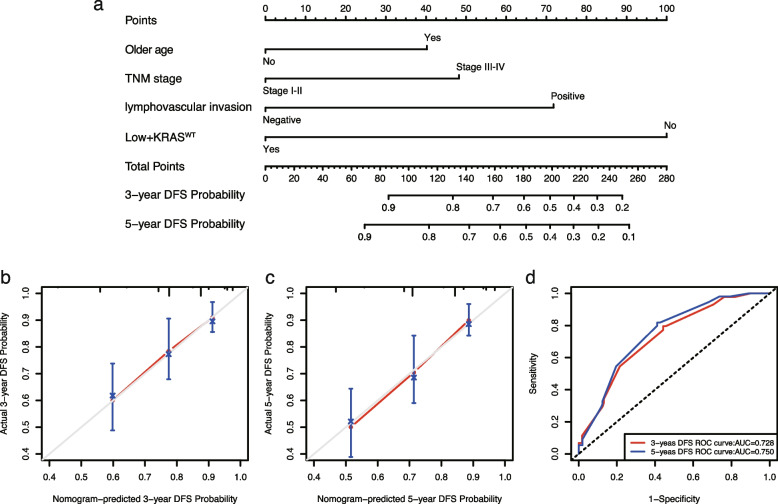
A nomogram, calibration curves and ROC curves for predicting DFS in CRC patients. **a**. A nomogram to predict 3-year and 5-year DFS based on model 6; (**b**, **c**). The calibration curves for predicting 3-year and 5-year DFS; **d**. ROC curves for 3-year and 5-year DFS

A nomogram for predicting 3-year and 5-year DFS was generated using the variables from model 6, including older age, TNM stage, lymphovascular invasion and Low + *KRAS*^WT^(Fig. 4a). The calibration curves for the nomogram were shown. The C-indexes of the nomogram for predicting 3-year and 5-year DFS were 0.712 and 0.713, respectively (Fig. 4b, c). ROC curve displays the performance of the nomogram. The AUCs of 3-years DFS and 5-years DFS were 0.728 and 0.750, respectively (Fig. 4d).

## Discussion

In this study, we found that *RIMS2* promoter showed a pattern of hypermethylation and low expression in snap-frozen CRC tissue comparing to normal tissue, which was consistent with the mechanism of tumor suppressor gene inactivation [30]. The findings that *RIMS2* expression may be subject to promoter methylation was further validated by 5-Aza-treatment. Then, we explored the prognosis value of the *RIMS2* methylation level in CRC. Patients with a high level of *RIMS2* methylation tended to have less frequent *KRAS* mutation and high differentiation. These patients also showed significantly worse postoperative outcome. Furthermore, a combination of *RIMS2* promoter methylation and *KRAS* status could predict the DFS better.

As a presynaptic protein, *RIMS2* plays an important role in normal neurotransmitter release [31]. Previous studies showed that *RIMS2* was mainly involved in some nervous system diseases, such as schizophrenia, heroin addiction, and Autism Spectrum Disorder [32–34]. Only limited evidence indicated that *RIMS2* may also contribute to the development of cancer [21]. However, RIMS1, another member of the RAS gene superfamily with similar structure and function to *RIMS2*, has shown a promising prognostic value in several kinds of cancer, like gastric cancer [35–38]. In addition, some studies found that dysregulation of Rab3, the interacting protein of *RIMS2*, may have a significant role in carcinogenesis and progression of CRC [39, 40]. As far as we know, this is the first study investigating the role of *RIMS2* in CRC. This study found that *RIMS2* methylation level was significantly higher in tumor compared to normal tissue, which results in low *RIMS2* expression in CRC. Furthermore, patients with higher methylation levels of *RIMS2* tended to have a worse outcome, indicating that *RIMS2* played an important role in the occurrence and progression of CRC. In this study, the association between *RIMS2* promoter methylation and prognosis of CRC remained significant after adjustment for some clinicopathological predictors, which showed a promising prognostic value of *RIMS2* promoter methylation in CRC. The expression of *RIMS2* was silenced by promoter methylation in CRC, indicating that *RIMS2* may be a potential epigenetic therapeutic target. As a biomarker, *RIMS2* promoter methylation can be easily detected by a PCR-based method, which allows it to be used broadly in clinical practice.

Still, there are some limitations in this study. The current study aimed to investigate new biomarkers and mainly focused on the prognostic value of *RIMS2* in CRC. However, the specific mechanism of *RIMS2* methylation in tumorigenesis and development remained unknown, which needed further confirmation by a series of experimental evidence. The influence of RIMS2 methylation on phenotypes of CRC cell lines will be valuable in studying the mechanism. Besides, patients were only from Chinese, and all the clinical data was obtained from the Sixth affiliated hospital of Sun Yat-Sen University. Multicenter, larger-scale studies would be helpful for confirming this result. In addition, some patients in cohort 2 whose molecular characteristics were incomplete may influence the study results. At last, as a retrospective study, the evidence level is insufficient and a prospective cohort is needed in future studies.

## Conclusion

*RIMS2* is frequently hypermethylated and lowly expressed in CRC, and patients with hypermethylated *RIMS2* tend to have poor survival outcomes. *RIMS2* promoter methylation is an independent prognostic biomarker for DFS in CRC.

## Data Availability

The data from the SYSU cohort that support the findings of this study are available on request from the corresponding author.

## Abbreviations

AIC: Akaike information criterion
AUC: Area under the ROC curve
CEA: Carcinoembryonic antigen
CRC: Colorectal cancer
DFS: Disease-free survival
FFPE: Formalin-fixed paraffin-embedded
LR: Likelihood ratio
MSI: Microsatellite instability
MSS: Microsatellite stability
NA: Not available
PMR: Percentage methylated reference
qMSP: Quantitative methylation-specific PCR
*RIMS2*: Regulating synaptic membrane exocytosis 2
ROC: Receiver operating characteristic curve

## References

[1] Sung H, Ferlay J, Siegel RL, Laversanne M, Soerjomataram I, Jemal A (2021). Global Cancer Statistics 2020: GLOBOCAN Estimates of Incidence and Mortality Worldwide for 36 Cancers in 185 Countries. CA Cancer J Clin.

[2] Xie Y, Shi L, He X, Luo Y (2021). Gastrointestinal cancers in China, the USA, and Europe. Gastroenterol Rep.

[3] Sveen A, Kopetz S, Lothe RA (2020). Biomarker-guided therapy for colorectal cancer: strength in complexity. Nat Rev Clin Oncol.

[4] Kocarnik JM, Shiovitz S, Phipps AI (2015). Molecular phenotypes of colorectal cancer and potential clinical applications. Gastroenterol Rep.

[5] Zou Q, Wang X, Ren D, Hu B, Tang G, Zhang Y (2021). DNA methylation-based signature of CD8+ tumor-infiltrating lymphocytes enables evaluation of immune response and prognosis in colorectal cancer. J Immunother Cancer.

[6] Okugawa Y, Grady WM, Goel A (2015). Epigenetic Alterations in Colorectal Cancer: Emerging Biomarkers. Gastroenterology.

[7] Chen Z, Huang Z, Luo Y, Zou Q, Bai L, Tang G (2021). Genome-wide analysis identifies critical DNA methylations within NTRKs genes in colorectal cancer. J Transl Med.

[8] Dor Y, Cedar H (2018). Principles of DNA methylation and their implications for biology and medicine. Lancet Lond Engl.

[9] Herman JG, Baylin SB (2003). Gene silencing in cancer in association with promoter hypermethylation. N Engl J Med.

[10] BLUEPRINT consortium. Quantitative comparison of DNA methylation assays for biomarker development and clinical applications. Nat Biotechnol. 2016;34:726–37. 10.1038/nbt.3605 27347756

[11] Vedeld HM, Goel A, Lind GE (2018). Epigenetic biomarkers in gastrointestinal cancers: The current state and clinical perspectives. Semin Cancer Biol.

[12] Wang Y, Südhof TC (2003). Genomic definition of RIM proteins: evolutionary amplification of a family of synaptic regulatory proteins. Genomics.

[13] Yasuda T, Shibasaki T, Minami K, Takahashi H, Mizoguchi A, Uriu Y (2010). Rim2α Determines Docking and Priming States in Insulin Granule Exocytosis. Cell Metab.

[14] Acuna C, Liu X, Südhof TC (2016). How to Make an Active Zone: Unexpected Universal Functional Redundancy between RIMs and RIM-BPs. Neuron.

[15] Gebhart M, Juhasz-Vedres G, Zuccotti A, Brandt N, Engel J, Trockenbacher A, et al. Modulation of Cav1.3 Ca2+ channel gating by Rab3 interacting molecule. Mol Cell Neurosci. 2010;44:246–59. 10.1016/j.mcn.2010.03.011 20363327

[16] Dulubova I, Lou X, Lu J, Huryeva I, Alam A, Schneggenburger R (2005). A Munc13/RIM/Rab3 tripartite complex: from priming to plasticity?. EMBO J.

[17] Fukuda M. Distinct Rab binding specificity of Rim1, Rim2, rabphilin, and Noc2. Identification of a critical determinant of Rab3A/Rab27A recognition by Rim2. J Biol Chem. 2003;278:15373–80. 10.1074/jbc.M212341200 12578829

[18] Ozaki N, Shibasaki T, Kashima Y, Miki T, Takahashi K, Ueno H (2000). cAMP-GEFII is a direct target of cAMP in regulated exocytosis. Nat Cell Biol.

[19] Ohara-Imaizumi M, Ohtsuka T, Matsushima S, Akimoto Y, Nishiwaki C, Nakamichi Y (2005). ELKS, a protein structurally related to the active zone-associated protein CAST, is expressed in pancreatic beta cells and functions in insulin exocytosis: interaction of ELKS with exocytotic machinery analyzed by total internal reflection fluorescence microscopy. Mol Biol Cell.

[20] Mukasa A, Ueki K, Ge X, Ishikawa S, Ide T, Fujimaki T (2004). Selective expression of a subset of neuronal genes in oligodendroglioma with chromosome 1p loss. Brain Pathol Zurich Switz.

[21] Tabariès S, McNulty A, Ouellet V, Annis MG, Dessureault M, Vinette M (2019). Afadin cooperates with Claudin-2 to promote breast cancer metastasis. Genes Dev.

[22] Shen D, Wang X, Wang H, Xu G, Xie Y, Zhuang Z (2022). Current Surveillance After Treatment is Not Sufficient for Patients With Rectal Cancer With Negative Baseline CEA. J Natl Compr Cancer Netw JNCCN.

[23] Yu H, Wang X, Bai L, Tang G, Carter KT, Cui J (2023). DNA methylation profile in CpG-depleted regions uncovers a high-risk subtype of early-stage colorectal cancer. J Natl Cancer Inst.

[24] Luo Y, Kaz AM, Kanngurn S, Welsch P, Morris SM, Wang J (2013). NTRK3 is a potential tumor suppressor gene commonly inactivated by epigenetic mechanisms in colorectal cancer. PLoS Genet.

[25] Eads CA, Danenberg KD, Kawakami K, Saltz LB, Blake C, Shibata D (2000). MethyLight: a high-throughput assay to measure DNA methylation. Nucleic Acids Res.

[26] Yu H, Bai L, Tang G, Wang X, Huang M, Cao G (2019). Novel Assay for Quantitative Analysis of DNA Methylation at Single-Base Resolution. Clin Chem.

[27] Vedeld HM, Merok M, Jeanmougin M, Danielsen SA, Honne H, Presthus GK (2017). CpG island methylator phenotype identifies high risk patients among microsatellite stable BRAF mutated colorectal cancers. Int J Cancer.

[28] Livak KJ, Schmittgen TD (2001). Analysis of relative gene expression data using real-time quantitative PCR and the 2(-Delta Delta C(T)) Method. Methods San Diego Calif.

[29] Hothorn T, Zeileis A (2008). Generalized maximally selected statistics. Biometrics.

[30] Esteller M (2002). CpG island hypermethylation and tumor suppressor genes: a booming present, a brighter future. Oncogene.

[31] Kaeser PS, Deng L, Fan M, Südhof TC (2012). RIM genes differentially contribute to organizing presynaptic release sites. Proc Natl Acad Sci U S A.

[32] Weidenhofer J, Scott RJ, Tooney PA (2009). Investigation of the expression of genes affecting cytomatrix active zone function in the amygdala in schizophrenia: effects of antipsychotic drugs. J Psychiatr Res.

[33] Nielsen DA, Ji F, Yuferov V, Ho A, He C, Ott J (2010). Genome-wide association study identifies genes that may contribute to risk for developing heroin addiction. Psychiatr Genet.

[34] Fan Y, Du X, Liu X, Wang L, Li F, Yu Y (2018). Rare Copy Number Variations in a Chinese Cohort of Autism Spectrum Disorder. Front Genet.

[35] Dai J, Li Z-X, Zhang Y, Ma J-L, Zhou T, You W-C (2019). Whole Genome Messenger RNA Profiling Identifies a Novel Signature to Predict Gastric Cancer Survival. Clin Transl Gastroenterol.

[36] Lv T, Miao Y-F, Jin K, Han S, Xu T-Q, Qiu Z-L (2018). Dysregulated circular RNAs in medulloblastoma regulate proliferation and growth of tumor cells via host genes. Cancer Med.

[37] Yang J, Hou Z, Wang C, Wang H, Zhang H (2018). Gene expression profiles reveal key genes for early diagnosis and treatment of adamantinomatous craniopharyngioma. Cancer Gene Ther.

[38] Maeda M, Yamashita S, Shimazu T, Iida N, Takeshima H, Nakajima T (2018). Novel epigenetic markers for gastric cancer risk stratification in individuals after Helicobacter pylori eradication. Gastric Cancer Off J Int Gastric Cancer Assoc Jpn Gastric Cancer Assoc.

[39] Chang Y-C, Su C-Y, Chen M-H, Chen W-S, Chen C-L, Hsiao M (2017). Secretory RAB GTPase 3C modulates IL6-STAT3 pathway to promote colon cancer metastasis and is associated with poor prognosis. Mol Cancer.

[40] Luo Y, Ye G-Y, Qin S-L, Mu Y-F, Zhang L, Qi Y (2016). High expression of Rab3D predicts poor prognosis and associates with tumor progression in colorectal cancer. Int J Biochem Cell Biol.

